# Actions of Allergens and Mediators in Allergy

**DOI:** 10.1155/2014/207345

**Published:** 2014-04-13

**Authors:** Shaoheng He, Huiyun Zhang, Peisong Gao

**Affiliations:** ^1^Allergy and Clinical Immunology Research Centre, Hospital of Liaoning Medical University, No. 2, Section 5, Renmin Street, Guta District, Jinzhou, Liaoning 121001, China; ^2^Department of Pathophysiology, Hainan Medical College, Haikou, Hainan 571101, China; ^3^Johns Hopkins Asthma and Allergy Center, Johns Hopkins University School of Medicine, Baltimore, MD 21224, USA

It has long been recognized that allergic inflammation is the fundamental pathological changes of allergy. Soluble allergens, specific IgE (sIgE), and mast cells or basophils are three key factors of allergy, representing causative factors, messenger, and primary effector cells, respectively. Eosinophils and neutrophils are known as secondary effector cells. In recent years, varieties of novel allergens and molecules involved in the allergic inflammation have been discovered, which makes the current understanding of the mechanisms of allergy even more confusing. We still have long way to go before the clear picture of pathogenesis of allergic inflammation can be accomplished, but our continuous and hard effort will help us to finally reach our destination drawing a complete picture of allergy. In this respect, this special issue will add a few new points in the picture of allergic inflammation.

Cockroach allergy has been associated with the development of asthma and recognized as a risk factor for emergency room admission of asthmatic patients, especially among inner city children living in low-income houses infested with cockroaches [1]. In the special issue, American cockroach allergen Per a 5 has been recognized as a minor allergen for American cockroach. Since its secondary protein structure is obtained and recombinant Per a 5 is generated, it will be a useful tool for studying the role of Per a 5 in cockroach allergy. Moreover, it is discovered that aryl hydrocarbon receptor (AhR) expression is higher in airway fibroblasts from asthmatic subjects, particularly when fibroblasts are treated with cockroach extract. An AhR agonist, 2,3,7,8-tetrachlorodibenzo-p-dioxin, can significantly enhance cockroach extract induced TGF*β*1 production in fibroblasts. These results suggest the role of AhR in modulating cockroach allergen induced immune responses through controlling the active TGF*β*1 release.

Classification of allergens in food plants and pollens is never easy. By using sequence alignment, progressive clustering, and comparison of 3-dimensional structures, Y. He and colleagues classify major allergens into 21 representative allergen groups, which are further divided into seven structural classes, each of which contains similar structural components. The grouping of allergens may help to understand the cross-reactivity between allergens.

Unlike studies on eosinophils and neutrophils alone, the interactions between these two secondary effector cells of allergy are relatively less investigated. Since it is an essential issue for understanding pathogenesis of allergic inflammation, it is examined by R. A. Luz and colleagues. Significant recruitment of eosinophils, neutrophils, and monocytes/macrophage by eotaxin is found in BALB/c mice, which can be abolished by eotaxin neutralization and 5-lipoxygenase-activating protein inhibitor MK886, suggesting that eotaxin induced recruitment of eosinophils is the initial event, and then followed by a 5-lipoxygenase dependent secondary neutrophil accumulation.

Inflamm-aging indicates the chronic inflammatory state resulting from increased secretion of proinflammatory cytokines and mediators such as IL-6 in the elderly. It is found that IL-6 protein production in cultured stromal cells from aged mouse spleen is significantly high compared to young mice upon LPS stimulation, which suggests that stromal cells may contribute to the chronic inflammatory condition such as allergic inflammation in the elderly.

As reviewed by P. Tripathi and colleagues, over the last few years, a significant progress has been made in understanding the role of a disintegrin and metalloproteinase 33 (ADAM33) in asthma. ADAM33 may play a role in airway remodeling because of its high expression in epithelium, myofibroblasts, and airway smooth muscle cells and its role in promoting angiogenesis and stimulating cell proliferation and differentiation. Molecules involved in allergic inflammation also include importins and exportins. A. Aggarwal and D. K. Agrawal described that the classical nuclear export signals are found on many transcription factors and molecules that are involved in the pathogenesis of allergic diseases. In addition, several immune modulators, including corticosteroids and vitamin D, elicit their cellular responses by regulating the expression and activity of importin molecules. In their review article, they provide also a comprehensive list of importin and exportin molecules and their specific cargo that are shuttled between cytoplasm and the nucleus. The review on the role and regulation of specific importin and exportin involved in the transport of activated transcription factors in allergic diseases will enable us to understand further cell signaling pathways in allergic inflammation.

*Shaoheng He* *Shaoheng He* *Huiyun Zhang* *Huiyun Zhang* *Peisong Gao* *Peisong Gao*
